# Seed Treatment with Illite Enhanced Yield and Nutritional Value of Soybean Sprouts

**DOI:** 10.3390/molecules27041152

**Published:** 2022-02-09

**Authors:** Man-Chul Ha, Dong-Young Im, Hung-Soo Park, Sanjeev Kumar Dhungana, Il-Doo Kim, Dong-Hyun Shin

**Affiliations:** 1 AOS Co. Ltd., Yongsan-myeon, Yeongdong-gun 29108, Korea; mcha@aosfnc.com (M.-C.H.); hspark@aosfnc.com (H.-S.P.); 2 Yeongdong County Office, Yeongdong-eup, Yeongdong-gun 29140, Korea; gidoo11@korea.kr; 3 Department of Southern Area Crop Science, National Institute of Crop Science, Rural Development Administration, Miryang 50424, Korea; sanjeevdhungana@yahoo.com; 4 International Institute of Research & Development, Kyungpook National University, Daegu 41566, Korea; ildookim@hanmail.net; 5 School of Applied Biosciences, Kyungpook National University, Daegu 41566, Korea

**Keywords:** antioxidant potential, nutrient, illite powder, soybean sprout, yield

## Abstract

Soybean sprouts, a nutritional food product, can contribute to food security because they can be grown within a week and do not require sophisticated technology. The yield and quality of soybean sprouts are influenced by various factors, including seed priming and growing conditions. The objective of this study was to investigate the effects of seed soaking in different concentrations of illite, a clay mineral, on the yield and quality of soybean sprouts. Soybean seeds soaked in five concentrations (0.5%, 1%, 3%, 5%, and 10%, *w*/*v*) of illite or tap water for 8 h were named IP-0.5, IP-1, IP3, IP-5, IP-10, and control, respectively. The highest sprout yield was found in IP-3, followed by IP-1, and IP-5, which had 11.1%, 8.8%, and 7.4% increments, respectively, compared to the control. The content of vitamin C, mineral element, isoflavone, total polyphenol, and total flavonoid was higher in many of the illite-treated soybean sprouts than in the control. The overall results indicated that pre-soaking soybean seeds in lower concentrations (0.5−3%, *w*/*v*) of illite could be helpful to enhance the yield and nutritional value of soybean sprouts in an easy and inexpensive way.

## 1. Introduction

Soybean (*Glycine max* L.) is an economically important crop with versatile end uses [1,2]; serving as an oil seed crop, feed for livestock, food for humans, and biofuel feedstock [3]. Germination enhances the availability of various nutrients such as vitamins, phytosterols, tocopherols, and isoflavones [4,5]. On the other hand, germination can remove or reduce several unwanted food constituents or their activities present in soybean seeds, including trypsin, chymotrypsin, lipoxygenase activity, phytic acid, and oligosaccharides [4,6,7]. Germination not only enhances nutritional and functional values, but also improves textural and organoleptic characteristics of legume seeds [8,9,10,11]. Soybean sprouts could help increase food security because it can be grown in a considerably short time, even without using advanced technologies. Soybean sprouts are an inexpensive food sources to supply dietary functional materials [12] and can also be used in producing cosmetic products with anti-aging and skin whitening effects [13].

Treatment of seeds before or during germination is a widely practiced approach to further enhance the value of seed sprouts. Various experiments on seed treatment, using biotic and/or abiotic factors, have been conducted to examine the effects on the quality of sprouts [14,15,16,17,18,19]. Among the abiotic factors of seed treatment, mineral-rich substances are one of the most commonly used seed treatment materials. Seed and sprout treatment with calcium chloride enhanced the yield and quality characteristics of soybean sprouts [20]. A zinc sulfate solution was applied to enrich the zinc content in soybean sprouts [21]. Natural mineral-rich water increased the mineral and quercetin content in Tartary buckwheat sprouts [22]. The selenium content in wheat, alfalfa, and sunflower sprouts was increased by treating the seeds with selenate [23]. Iron fortification with ferrous sulfate during germination increased the iron concentration in the germinated brown rice [24]. Brown rice treated with zinc sulfate during germination increased the amount and bioavailability of zinc [25]. The use of zinc solution improved the hygienic and nutritional properties of durum wheat sprouts [26].

Potassium (K), the most abundant cation comprising up to 10% on a dry weight basis, plays a vital role in plant growth and development [27]. Supplemental K significantly enhances the synthesis of osmolytes such as free proline, amino acids, and sugars in plants [28]. In addition, a large number of enzymes is entirely dependent on or regulated by K, influencing cell elongation and osmotic adjustment [29]. Application of K could increase primary and secondary metabolites including antioxidative compounds [30,31,32]. K increased total phenols, flavonoids, and antioxidant activity in tomato [33] and basil [34].

Illite is a clay mineral that contains various mineral elements like potassium, calcium, magnesium, silicon, iron, and aluminum [35,36,37]. Very limited studies on the effect of illite on the growth and development of plants have been conducted. Illite effectively improved the germination and growth of lettuce [38]. The crude oil, ash, calcium, free sugars, hyperoside, and isoquercitrin contents were increased in *Saururus chinensis* Baill [39]. Considering the mineral element composition and other benefits of illite in plant growth and development, this study aimed to investigate the effect of seed soaking with illite solutions on the growth and quality of soybean sprouts. This is the first study to investigate the influence of illite on soybean sprouts.

## 2. Results

### 2.1. Yield, Moisture Content, and Vitamin C Content

**Figure 1:**
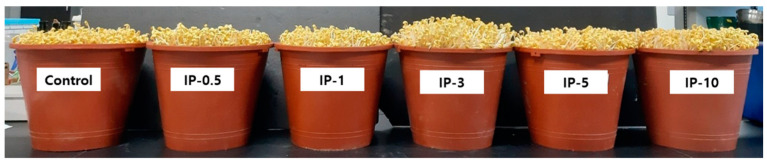
Soybean sprouts grown with different concentrations of illite. Samples are defined in Table 1.

Illite treatment significantly influenced the yield and vitamin C content of soybean sprouts; however, the moisture content of the sprouts was not affected (Table 1). The highest sprout yield increment was found for IP-3 (11.1%), followed by IP-1 (8.8%), IP-5 (7.4%), compared to the control (Figure 1 and Table 1). The vitamin C content was also significantly highest for IP-3 (18.21 mg/100 g fresh weight, FW). The control (16.22 mg/100 g FW) and IP-0.5 (16.62 mg/100 g FW) showed significantly equal vitamin C content. The results indicated that both high and low concentrations of illite treatment do not favor sprout yield increment and vitamin C content.

### 2.2. Color Value of Soybean Sprouts

Although the lightness value was unaffected, the redness value of all sprout samples was decreased and the yellowness value of IP-0.5 and IP-1 was increased by illite treatment (Table 2). The significantly lowest redness value was found for IP-0.5 (0.92), IP-1 (0.99), and IP-3 (0.98). However, IP-1 had significantly highest yellowness values.

### 2.3. Mineral Content

Illite treatment increased the total mineral content of soybean sprouts except in IP-3 (Table 3). The highest total mineral content was found in IP-1 (36,115.08 mg/kg), followed by IP-5 (36,113.07 mg/kg) compared to the control (35,425.68 mg/kg). K (16,547.21–17,354.05 mg/kg DW) and P (11,922.78–12,669.21 mg/kg DW) were the most abundant and Mn (21.23–27.31 mg/kg DW) and Cu (24.50–38.02 mg/kg DW) were the least abundant mineral elements in the sprout samples. Out of the nine minerals measured, the amount of six mineral elements—Ca, Cu, Fe, Mg, Mn, and P—were higher in one or more sprout samples treated with illite. In all the illite-treated soybean sprouts, the content of Na and Zn was reduced in comparison to the control. The amount of K was reduced in the sprout samples IP-3, IP-5, and IP-10, which were treated with higher concentrations of illite.

### 2.4. Isoflavone Content

The total isoflavone content of soybean sprouts was increased with the concentration of illite solution (Table 4). The amount of daidzin, daidzein, genistin, and glycitin were significantly increased in all the illite-treated sprouts whereas that of glycitein and genistein was reduced by illite treatment. Daidzin (249.47–319.67 mg/kg DW) and genistin (127.89–208.23 mg/kg DW) were the most abundantly found isoflavones whereas daidzein (10.85–24.80 mg/kg DW) glycitein (8.12–13.75 mg/kg DW) were the least abundant isoflavones in the sprout samples.

### 2.5. DPPH, Total Polyphenol and Flavonoid Contents, and SOD-like Activity

Illite treatment significantly increased the antioxidant potentials of most of the soybean sprout samples (Table 5). The 1,1-diphenyl-2-picrylhydrazyl (DPPH) free radical scavenging potential was significantly highest in IP-1 (85.90%), followed by IP-3 (80.90%). The lowest DPPH inhibition was observed with IP-0.5 (58.08%) which was statistically equal with the control (60.02%). The superoxide dismutase (SOD)-like activity was significantly highest in IP-10 (41.81%), followed by IP-3 (37.63%) which was statistically equal to that in IP-5 (36.80%). IP-10 had the highest total polyphenol content and SOD-like activity. The greatest amount of total flavonoid content was found in IP-5 (777.00%) which was statistically equal to that found in IP-10 (764.14%).

### 2.6. Free Amino Acid Composition

The essential and total amino acid contents in the soybean sprouts were increased with 0.5% illite treatment, however, were reduced with the higher concentrations of illite (Table 6). In the case of non-essential amino acid, all concentrations of illite treatment (4.45–5.34 mg/g DW) decreased the amino acid content compared to the control (5.54 mg/g DW). A total of 8 essential, 8 non-essential, and 14 other free amino acids were detected in all samples. Seven free amino acids were not detectable in the sprout samples. The amount of six essential, one non-essential, and one other free amino acids were significantly higher in at least one illite-treated sprout sample.

## 3. Discussion

Mineral elements, including potassium and calcium, present in illite might have influenced the growth-promoting substances, thereby increased the yield and nutrient content of illite-treated soybean sprouts. The positive role of potassium on plant growth and development has been well documented [27,28]. Potassium plays role in the regulation of a large number of enzymes, cell elongation, and osmotic adjustment [29]. Calcium and other minerals [35,36,37] might have improved the levels of plant hormones indoleacetic acid and gibberellin, which increase the yield and vitamin C content in the soybean sprouts [20]. A similar result of enhanced growth of tomato was observed in the plants grown in illite-containing soil [37].

Color of a food product plays an important role to attract consumers to pay for the product [40]. Illite treatment did not reduce the lightness and yellowness values, which are some of the desirable characteristics of soybean sprouts [41]. Moreover, the lightness value was increased in IP-0.5 and IP-1. Although the physiological mechanisms of color variations in sprouts were not clear, illite treatment could enhance the soybean sprout color.

It can be expected that the increment in mineral content of sprouts resulted from the uptake of minerals present in illite and possible subsequent physiochemical mechanisms as reported in the previous studies with zinc sulfate-applied soybean sprouts [21,42], cultivation of tartary buckwheat and wheat sprouts in mineral-rich water [22], and selenium-treated cereal sprouts [23]. Elements—including Fe, Cu, Ca, and Mg—which were increased in some illite-treated sprouts, are most commonly lacking in human diets [43]. Minerals Mg, K, and Ca are advantageous against hypertension [44]; Fe is beneficial in oxygen transport, energy metabolism, mitochondrial respiration, DNA synthesis, and cellular growth and differentiation [45]. Similarly, Zn contributes to growth, development, differentiation, DNA synthesis, RNA transcription, and cellular apoptosis [46]. The increased mineral content in soybean sprouts with illite treatment indicated the effectiveness of illite to improve the mineral content of sprouts.

Similar results of increased total isoflavone content were found in soybean sprouts with calcium treatment [20], although the exact mechanism was not clear. One of the reasons for the improved isoflavone content could be due to increased phenylalanine and isoflavone synthetase activities [20,47] owing to the effect of mineral-rich illite treatment. Soybean seed soaking with a mineral-rich Pu-erh tea also enhanced the isoflavone content of soybean sprouts [48]. Soy isoflavones are known for their health benefits against various disorders. Results showed that illite can effectively enhance the isoflavones content in soybean sprouts.

Illite has shown antioxidant and antibacterial activities because of the abundance of important clay elements in its structure [49]. The elevated antioxidant potential of the illite-treated sprouts might be owing to the abundance of elements such as calcium and potassium [34,35,36,37] and/or the antioxidative effect of illite [49]. The antioxidant activity in tomato leaves was supposed to be increased by illite treatment [37]. The nutraceutical component—including polyphenolic compounds, flavonoids, vitamin C, and the antioxidant capacity in the hydroponically grown basil—was increased with added potassium [34]. Production of excessive reactive oxygen species (ROS) results in oxidative damage in lipids, proteins, and DNA [50]. Elevated levels of the ROS may harm cells by lipids peroxidation, proteins oxidation, nucleic acid destruction, enzyme inhibition, the programmed cell death activation pathway, and eventually cell death [51,52]. Antioxidants, including phenolics and flavonoids, scavenge ROS and protect cells from potential harm [53,54,55]. Thus, illite treatment was found useful in increasing the amount of total polyphenols and flavonoids, resulting in the production of antioxidant-rich soybean sprouts.

The reduced amount of amino acids, especially in the higher concentrations of illite treatment, might be due to degradation of seed protein for sprout growth and synthesis of bioactive compounds [56]. We could not explain the exact reasons for the inconsistent variations in some of the nutrient components, including amino acids, among different concentrations of illite treatment. We speculated that the higher concentrations of illite might have imposed some kind of stress resulting in reduced amino acid content. The calcium present in illite might have played a role in the activation of diamine oxidase activity and thereby increased content of some amino acids in the illite-treated sprouts [20].

## 4. Materials and Methods

### 4.1. Experiment Materials and Reagents

Soybean (*Glycine max* L.) cv. Seoritae, a landrace with medium seed size (40 g/100 seeds), was obtained from Nonghyup (Daegu, Korea). Illite powder was obtained from Yeongdong-gun, Chungcheong-bukdo, Korea. The following chemicals and reagents were obtained for the present study: 1,1-diphenyl-2-picrylhydrazyl (DPPH), Folin–Ciocalteau reagent, isoflavone standards (≥95% purity, Sigma-Aldrich Corporation, St. Louis, MO, USA), dimethyl sulfoxide (DMSO), and pyrogallol (Sigma-Aldrich Corporation, St. Louis, MO, USA); amino acid standards (Wako Pure Chemical Industries, Ltd., Osaka, Japan). All the other chemicals were of analytical grade.

### 4.2. Cultivation of Soybean Sprouts and Measurement of Sprout Yield

Soybean sprouts were cultivated for 6 days following the method described by Kim et al. [12] with some modifications. One kilogram of intact seeds for five illite treatments and one control with three replications each were washed with tap water and excess water was drained off. The seeds were surface-dried at room conditions and were soaked in tap water containing five different concentrations (0.5%, 1%, 3%, 5%, and 10% *w*/*v*) of illite powder or tap water alone for 8 h. After soaking, the seeds were thoroughly washed with tap water to rinse the illite particles adsorbed on the seed surface and were kept in 15-L plastic buckets with a perforated base for sprout cultivation. The samples were named as control, IP-0.5, IP-1, IP-3, IP-5, and IP-10 for the seeds soaked in tap water alone, 0.5%, 1%, 3%, 5%, and 10% (*w*/*v*) illite solutions prepared in tap water, respectively. Soybean sprouts were grown at room temperature (22 ± 2 °C).

The yield of soybean sprouts was measured by deducting the weight of the empty bucket from the gross weight of each bucket containing sprouts [12].

### 4.3. Measurement of Moisture Content and Preparation of Sprout Powder

The moisture content of soybean sprouts was determined using the oven-dry method following the method of AOAC [57] as described by Kim et al. [48].

For the preparation of sprout sample powder, the freshly harvested sprouts were kept at −70 °C for 24 h before being subjected to freeze-drying. The freeze-dried sprouts were ground into powder using a commercial grinder (HIL-G-501, Hanil Co., Seoul, Korea). The powdered samples were kept in airtight sample bottles and stored at −20 °C until analyses.

### 4.4. Determination of Vitamin C Content

The vitamin C content of sprouts was determined following a standard method [58]. Five grams of sample powder was mixed with 7.5 mL of 3% metaphosphoric acid solution and homogenized (AM-8, Nihonseike Kaisha, Tokyo, Japan), followed by the addition of 12.5 mL of the acid solution and filtration. Six milliliters of the filtrate were titrated with 0.025% of 2,6-dichloroindophenol. In this reaction, the vitamin C contained in the extract is oxidized and the indophenol dye reduces to a colorless compound.

### 4.5. Color Measurement of Soybean Sprouts

The L* (lightness), a* (redness, + or greenness, −), and b* (yellowness, + or blueness, −) values of sprout powder were determined using a Chroma meter (CR-300, Minolta Corp., Osaka, Japan). A Minolta calibration plate (YCIE = 94.5, XCIE = 0.3160, YCIE = 0.330) and a HunterLab standard plate (L* = 97.51, a* = −0.18, b* = +1.67) were used to standardize the instrument using a D65 illuminant [59].

### 4.6. Determination of Free Amino Acid Content

The free amino acid profile was determined following the method of Je et al. [60] with some modifications. Sprout powder (1 g) was hydrolyzed with 6 N hydrochloric acid (10 mL) in a sealed-vacuum ampoule at 110 °C for 24 h. The acid was removed from the hydrolyzed sample using a rotary evaporator and the content was mixed with 0.2 M sodium citrate buffer (pH 2.2) to make the final volume 5.0 mL. The mixture was passed through a C-18 Sep Pak (Waters Co., Milford, MA, USA) cartridge and filtered through a 0.22 μm membrane filter (Millipore, Billerica, MA, USA). The amino acids were determined using an automatic amino acid analyzer (Biochrom-20, Pharmacia Biotech Co., Uppsala, Sweden).

### 4.7. Determination of Mineral Content

The mineral content of sprouts was measured following the procedure described by Skujins [61] with some modifications. A half gram of sprout powder was mixed with 15 mL nitric acid in a cup, followed by a dilution with an equal volume of distilled water. The concentration of mineral elements was measured using an inductively coupled plasma atomic emission spectrometer (ICP AES: Varian Vista, Varian Australia, Victoria, Australia).

### 4.8. Measurement of Isoflavone Content

Two hundred milligrams of sprout powder were extracted with 6.0 mL methanol (80%) by adopting an ultrasonic-assisted method (40 °C, 30 min), followed by centrifugation (13,000 rpm, 10 min). The supernatant was filtered through a 0.45 µm membrane filter (Millipore Corp., Bedford, MA, USA) before being subjected to high-performance liquid chromatography (HPLC) analysis. The isoflavone content was determined using an HPLC system [62] under the following conditions: flow rate 1 mL/min; the mobile phase: solvent A—aqueous acetic acid (0.1%), and solvent B—acetic acid in acetonitrile (0.1%). HPLC running condition consisted of a gradient of 13–35% B during a 52 min period, oven temperature of 35 °C, and an injection volume of 20 µL. The eluted isoflavones were detected at 260 nm. Each peak was identified by the retention time and the characteristic UV spectrum in comparison with the corresponding standards.

### 4.9. Determination of DPPH Free Radical Scavenging Potential

One gram sprout powder was extracted with 10 mL of absolute methanol using a shaking incubator (150 rpm, 25 °C) for 8 h, followed by centrifugation at room temperature (3000 rpm, 15 min) and filtration through a 0.2-µm syringe filter (Waters Co., Milford, MA, USA). One hundred microliters of sample extract and 0.1% (*w*/*v*) methanolic solution of DPPH each were mixed in microplate wells and then incubated at room temperature for 30 min under dark conditions. The absorbance value of reaction mixtures was measured at 517 nm using a microplate spectrophotometer (Multiskan GO, Thermo Fisher Scientific, Vantaa, Finland). The DPPH radical-scavenging potential of sprouts was assayed following the method described by Blois [63] and Dhungana et al. [64].

### 4.10. Determination of Total Polyphenol Content

The sample extracts prepared for DPPH assay were also used in determining the total polyphenol content (TPC) following the Folin–Ciocalteau method [60] as described earlier [65]. Fifty microliters of the sample extracts and 1000 μL of 2% (*w*/*v*) aqueous sodium carbonate were mixed in microtubes and left for 3 min. After 3 min, 50 μL of 1 N Folin–Ciocalteau reagent was added to the mixture and incubated at room temperature for 30 min under dark conditions. The absorbance value of reaction mixtures was measured at 750 nm using a microplate spectrophotometer (Multiskan GO; Thermo Fisher Scientific, Vantaa, Finland). Gallic acid (GA) was used as the standard to plot the calibration curve and the TPC content of sprout samples was determined as GA equivalent (GAE).

### 4.11. Total Flavonoid Content Analysis

The total flavonoid content (TFC) of sprouts was estimated following the method described by Zhishen et al. [66] with some modifications. The methanolic sample extract that was prepared for the DPPH and total polyphenol assays was used in this TFC analysis too. One hundred microliters sample extracts, 500 μL methanol, 50 μL 10% aluminum chloride, 50 μL 1 M hydrochloric acid, and 300 μL distilled water were mixed in microtubes and then incubated at room temperature for 30 min under dark conditions. Two hundred microliters of reaction mixtures were put into 96-well plates and the absorbance value was measured at 510 nm using a microplate spectrophotometer (Multiskan GO; Thermo Fisher Scientific, Vantaa, Finland). The TFC was determined by using the calibration curve plotted using quercetin (QE) as a standard.

### 4.12. Determination of Superoxide Dismutase (SOD)-Like Activity

The SOD-like activities of the sprout samples were determined following the analysis procedures of Debnath et al. [67] and Adhikari et al. [68]. A half-gram sprout powder was homogenized with 5 mL phosphate extraction buffer (pH 7.8) using an ice-cooled mortar and pestle. The mixture was centrifuged (15,000 rpm, 20 min). One hundred microliters supernatant, 1300 μL Tris-HCl buffer (50 mM Tris, 10 mM EDTA, pH 8.5), and 100 μL 7.2 mM pyrogallol were mixed and incubated at room temperature for 10 min under dark conditions. After 10 min of incubation, the reaction was terminated by mixing 50 μL 1 N hydrochloric acid into the reaction mixture. The amount of pyrogallol oxidized during the reaction was estimated by measuring the absorbance value of the mixture at 420 nm using a microplate spectrophotometer (Multiskan GO, Thermo Fisher Scientific, Vantaa, Finland).

### 4.13. Statistical Analysis

Data were subjected to analysis of variance using SAS 9.4 (SAS Institute, Cary, NC, USA). The significant differences between treatment means were analyzed using Tukey test (*p* < 0.05). Average values of triplicate measurements were considered for statistical analysis unless otherwise mentioned specifically in any assays.

## 5. Conclusions

The effects of illite treatment on soybean sprouts were examined considering the yield and nutritional values of the sprouts. The yield, vitamin C content, mineral content, isoflavone content, antioxidant potentials, total polyphenol, and flavonoid of many of the illite-treated soybean sprouts were higher than those of the control. Overall, soaking of soybean seeds in lower concentrations (0.5−3%, *w*/*v*) of illite could be an effective and efficient way to improve the yield and nutritional values of soybean sprouts.

## Figures and Tables

**Table 1:** Effect of different concentrations of illite treatment on the yield, moisture content, and vitamin C content of soybean sprouts cultivated for 6 days.

Sample ^1^	Total Weight (g)	Moisture (%)	Vitamin C (mg/100 g Fresh Weight)
Control	5525 ± 30 d ^2^ (100.0%)	87.66 ± 0.02 a	16.22 ± 0.21 c
IP-0.5	5892 ± 22 c (106.6%)	87.23 ± 0.11 a	16.62 ± 0.53 bc
IP-1	6012 ± 28 b (108.8%)	88.00 ± 1.02 a	16.98 ± 0.31 b
IP-3	6137 ± 33 a (111.1%)	87.63 ± 0.09 a	18.21 ± 0.30 a
IP-5	5933 ± 41 c (107.4%)	88.02 ± 1.00 a	16.82 ± 0.31 b
IP-10	5899 ± 18 c (106.7%)	88.01 ± 0.51 a	16.90 ± 0.09 b

^1^ Control, soybean seeds soaked in tap water for 8 h; IP-0.5, soybean seeds soaked in tap water containing 0.5% (*w*/*v*) illite powder for 8 h; IP-1, soybean seeds soaked in tap water containing 1.0% (*w*/*v*) illite powder for 8 h; IP-3, soybean seeds soaked in tap water containing 3.0% (*w*/*v*) illite powder for 8 h; IP-5, soybean seeds soaked in tap water containing 5.0% (*w*/*v*) illite powder for 8 h; IP-10, soybean seeds soaked in tap water containing 10.0% (*w*/*v*) illite powder for 8 h. Percentage, for total weight, in parentheses denotes the variation in sprout yields with respect to the control. ^2^ Values are expressed as mean ± standard deviation of three replicates. Values followed by different letters in the same column indicate significant difference (*p* < 0.05, Tukey test).

**Table 2:** Hunter’s color values of soybean sprouts grown after different concentrations of illite treatment.

Sample ^1^	Color Value ^2^		
	L* (Lightness)	a* (Redness)	b* (Yellowness)
Control	76.33 ±0.91 a ^3^	1.72 ± 0.04 a	20.06 ± 0.46 d
IP-0.5	77.12 ± 0.82 a	0.92 ± 0.03 c	22.01 ± 0.28 b
IP-1	76.65 ± 0.72 a	0.99 ± 0.05 c	23.62 ± 0.32 a
IP-3	77.03 ± 0.49 a	0.98 ± 0.03 c	22.33 ± 0.23 b
IP-5	76.77 ± 0.69 a	1.20 ± 0.04 b	20.57 ± 0.07 d
IP-10	76.99 ± 0.88 a	1.16 ± 0.06 b	20.99 ± 0.08 c

^1^ Samples are defined in Table 1. ^2^ L*, lightness (100, white; 0, black); a*, redness (−, green; +, red); b*, yellowness (−, blue; +, yellow). ^3^ Values are expressed as mean ± standard deviation of three replicates. Values followed by different letters in the same column are significantly different (*p* < 0.05, Tukey test).

**Table 3:** Mineral contents (mg/kg of dry weight) of soybean sprouts cultivated after different concentrations of illite treatment.

Element	Sample ^1^					
	Control	IP-0.5	IP-1	IP-3	IP-5	IP-10
Ca	3032.32 ± 46.13 d ^2^	3054.27 ± 59.59 c	3209.42 ± 37.14 b	3286.27 ± 44.66 b	3661.19 ± 48.71 a	3660.18 ± 27.26 a
Cu	35.21 ± 0.37 c	29.28 ± 6.12 e	30.97 ± 0.17 d	24.50 ± 0.23 f	38.02 ± 0.16 a	36.29 ± 0.19 b
Fe	85.41 ± 0.23 d	84.18 ± 0.43 e	83.56 ± 0.32 e	89.38 ± 0.50 c	104.55 ± 0.45 b	110.23 ± 0.37 a
K	17,354.05 ± 146.55 a	17,181.56 ± 193.58 a	17,287.40 ± 121.60 a	16,547.21 ± 134.75 c	16,961.22 ± 197.71 b	16,661.23 ± 161.61 c
Mg	2317.08 ± 38.19 b	2271.01 ± 34.82 c	2374.51 ± 28.24 a	2242.94 ± 19.60 c	2251.99 ± 10.37 c	2260.11 ± 19.23 c
Mn	25.87 ± 0.06 b	27.31 ± 0.21 a	25.88 ± 0.08 b	25.27 ± 0.16 b	21.23 ± 0.12 d	23.25 ± 0.31 c
Na	595.16 ± 2.85 a	379.36 ± 4.97 f	474.07 ± 4.63 b	391.87 ± 4.31 e	423.36 ± 6.73 d	439.21 ± 5.51 c
Zn	57.80 ± 0.15 a	44.94 ± 0.15 c	45.25 ± 0.06 b	42.75 ± 0.23 d	42.15 ± 0.09 e	42.55 ± 0.08 d
P	11,922.78 ± 33.27 c	12,583.29 ± 51.77 a	12,584.02 ± 77.32 a	12,197.63 ± 35.23 b	12,609.36 ± 20.47 a	12,669.21 ± 19.27 a
Total	35,425.68	35,655.20	36,115.08	34,847.82	36,113.07	35,902.26

^1^ Samples are defined in Table 1. ^2^ Values are expressed as mean ± standard deviation of two replicates. Values followed by different letters in the same row are significantly different (*p* < 0.05, Tukey test).

**Table 4:** Isoflavone content (mg/kg dry weight) of soybean sprouts cultivated after different concentrations of illite treatment.

Isoflavone	Sample ^1^					
	Control	IP-0.5	IP-1	IP-3	IP-5	IP-10
Daidzin	249.47 ± 1.32 e ^2^	264.35 ± 2.93 d	284.78 ± 5.19 c	292.12 ± 2.37 c	302.89 ± 5.96 b	319.67 ± 4.00 a
Daidzein	10.85 ± 0.98 f	12.82 ± 0.60 e	16.58 ± 0.75 d	18.12 ± 0.70 c	20.45 ± 1.34 b	24.80 ± 0.97 a
Genistin	127.89 ± 2.09 f	136.98 ± 3.47 e	161.48 ± 3.97 d	181.12 ± 1.27 c	190.04 ± 4.96 b	208.23 ± 4.05 a
Glycitin	52.49 ± 1.73 f	57.97 ± 2.23 e	69.71 ± 2.16 d	75.00 ± 1.14 c	86.80 ± 1.22 b	97.18 ± 2.07 a
Glycitein	13.75 ± 0.28 a	13.11 ± 0.19 b	11.39 ± 0.25 c	10.12 ± 0.11 d	9.27 ± 0.29 e	8.12 ± 0.44 f
Genistein	41.34 ± 1.68 a	39.10 ± 0.44 b	33.46 ± 1.20 c	30.15 ± 1.16 d	27.34 ± 1.13 e	21.95 ± 2.49 f
Total	495.79	524.33	577.40	606.63	636.79	679.95

^1^ Samples are defined in Table 1. ^2^ Values are expressed as mean ± standard deviation of two replicates. Values followed by different letters in the same row are significantly different (*p* < 0.05, Tukey test).

**Table 5:** 1,1-diphenyl-2-picrylhydrazyl (DPPH), total polyphenol and flavonoid contents, and superoxide dismutase (SOD)-like activity of soybean sprouts treated with different concentrations of illite.

Sample ^1^	DPPH (% Inhibition)	Total Polyphenol (μg GAE ^2^/g)	Total Flavonoid (μg QE ^3^/g)	SOD-Like Activity (% Inhibition)
Control	60.02 ± 1.04 d ^4^	587.66 ± 5.14 b	633.42 ± 6.23 e	27.60 ± 1.26 e
IP-0.5	58.08 ± 1.54 d	402.66 ± 13.42 e	697.28 ± 3.83 b	30.69 ± 0.55 d
IP-1	85.90 ± 1.23 a	417.65 ± 10.91 de	657.14 ± 9.55 d	33.25 ± 1.01 c
IP-3	80.90 ± 0.20 b	430.22 ± 11.24 d	689.27 ± 7.72 c	37.63 ± 1.13 b
IP-5	70.90 ± 0.50 c	456.94 ± 12.48 c	777.00 ± 5.92 a	36.80 ± 2.37 b
IP-10	71.32 ± 1.62 c	601.61 ± 8.88 a	764.14 ± 5.32 a	41.81 ± 1.34 a

^1^ Samples are defined in Table 1. ^2^ Gallic acid equivalent. ^3^ Quercetin equivalent. ^4^ Values are expressed as mean ± standard deviation of three replicates. Values followed by different letters in the same column are significantly different (*p* < 0.05, Tukey test).

**Table 6:** Free amino acid composition (mg/g of dry weight) of soybean sprouts cultivated after different concentrations of illite treatment.

Amino Acid	Sample ^1^					
	Control	IP-0.5	IP-1	IP-3	IP-5	IP-10
Essential Amino Acid						
l-Threonine	1.17 ± 0.02 b ^2^	1.25 ± 0.01 a	0.92 ± 0.01 d	1.13 ± 0.02 b	0.99 ± 0.02 c	0.98 ± 0.02 c
l-Valine	2.43 ± 0.02 b	2.88 ± 0.04 a	2.04 ± 0.03 d	2.46 ± 0.02 b	2.21 ± 0.03 c	2.22 ± 0.03 c
l-Methionine	0.15 ± 0.01 b	0.14 ± 0.02 b	0.10 ± 0.01 c	0.14 ± 0.01 b	1.18 ± 0.02 a	1.19 ± 0.04 a
l-Isoleucine	1.19 ± 0.03 a	1.29 ± 0.01 b	0.94 ± 0.02 d	1.10 ± 0.03 b	1.02 ± 0.02 c	1.04 ± 0.02 c
l-Leucine	0.59 ± 0.01 a	0.59 ± 0.01 a	0.43 ± 0.01 d	0.53 ± 0.01 b	0.48 ± 0.01 c	0.49 ± 0.02 c
l-Phenylalanine	1.86 ± 0.02 b	1.98 ± 0.03 a	1.35 ± 0.02 e	1.66 ± 0.03 c	1.48 ± 0.03 d	1.50 ± 0.03 d
l-Lysine	1.16 ± 0.01 b	1.48 ± 0.02 a	0.71 ± 0.01 e	0.94 ± 0.02 c	0.87 ± 0.01 d	0.88 ± 0.02 d
l-Histidine	1.69 ± 0.02 b	1.82 ± 0.03 a	1.29 ± 0.03 e	1.53 ± 0.04 c	1.46 ± 0.03 d	1.50 ± 0.01 c
Sub-total	10.24	11.43	7.78	9.49	9.69	9.80
Non-essential Amino Acid						
l-Asparitic acid	0.36 ± 0.01 ab	0.38 ± 0.02 a	0.36 ± 0.01 ab	0.31 ± 0.01 c	0.34 ± 0.01 b	0.35 ± 0.01 ab
l-Serine	1.85 ± 0.02 a	1.87 ± 0.04 a	1.46 ± 0.02 d	1.79 ± 0.01 b	1.62 ± 0.02 c	1.64 ± 0.03 c
l-Glutamic acid	0.08 ± 0.01 b	0.01 ± 0.01 c	0.09 ± 0.01 b	0.07 ± 0.01 b	0.17 ± 0.02 a	0.16 ± 0.01 a
Glycine	0.18 ± 0.02 a	0.15 ± 0.02 ab	0.13 ± 0.02 b	0.17 ± 0.02 a	0.13 ± 0.01 b	0.13 ± 0.02 b
l-Alanine	1.51 ± 0.02 a	1.40 ± 0.03 b	1.21 ± 0.03 c	1.45 ± 0.03 b	1.30 ± 0.02 c	1.29 ± 0.03 c
l-Tyrosine	0.14 ± 0.01 a	0.12 ± 0.02 ab	0.03 ± 0.01 c	0.12 ± 0.02 ab	0.10 ± 0.01 b	0.11 ± 0.02 ab
l-Arginine	0.91 ± 0.01 a	0.93 ± 0.03 a	0.82 ± 0.02 b	0.31 ± 0.01 d	0.52 ± 0.01 c	0.53 ± 0.01 c
Proline	0.51 ± 0.01 a	0.48 ± 0.03 a	0.35 ± 0.01 c	0.45 ± 0.01 b	0.40 ± 0.02 b	0.41 ± 0.02 b
Sub-total	5.54	5.34	4.45	4.67	4.58	4.62
Other Amino Acid						
*O*-Phospho-L-serine	0.15 ± 0.01 a	0.16 ± 0.01 a	0.11 ± 0.0.2 b	0.13 ± 0.02 ab	0.13 ± 0.02 ab	0.14 ± 0.01 a
Taurine	ND ^3^	ND	ND	ND	ND	ND
*O*-Phospho ethanol amine	ND	ND	ND	ND	ND	ND
Urea	0.76 ± 0.02 b	0.90 ± 0.01 a	0.62 ± 0.01 c	0.73 ± 0.01 b	0.72 ± 0.02 b	0.73 ± 0.03 b
l-Sarcosine	0.04 ± 0.01 a	0.01 ± 0.01 b	0.03 ± 0.01 ab	0.04 ± 0.01 a	0.04 ± 0.01 a	0.04 ± 0.01 a
l-α-Amino asipic acid	0.16 ± 0.01 ab	0.17 ± 0.01 a	0.12 ± 0.01 c	0.15 ± 0.02 ab	0.14 ± 0.02 ab	0.14 ± 0.01 b
l-Citrulline	0.05 ± 0.01 a	0.05 ± 0.01 a	0.04 ± 0.01 a	0.04 ± 0.01 a	0.06 ± 0.01 a	0.05 ± 0.01 a
l-α-Amino-n-butyric acid	0.09 ± 0.01 a	0.09 ± 0.01 a	0.07 ± 0.01 a	0.09 ± 0.01 a	0.08 ± 0.02 a	0.08 ± 0.01 a
l-Cystine	0.08 ± 0.01 a	0.08 ± 0.01 a	0.08 ± 0.01 a	0.08 ± 0.01 a	0.07 ± 0.01 a	0.07 ± 0.01 a
Cystathionine	0.02 ± 0.01 a	0.01 ± 0.01 a	0.01 ± 0.01 a	0.03 ± 0.01 a	0.02 ± 0.01 a	0.02 ± 0.01 a
β-Alanine	0.27 ± 0.02 a	0.28 ± 0.02 a	0.22 ± 0.01 b	0.25 ± 0.03 ab	0.24 ± 0.03 ab	0.23 ± 0.01 b
d,l-β-Amino isobutyric acid	0.12 ± 0.02 a	0.12 ± 0.01 a	0.12 ± 0.03 a	0.11 ± 0.01 a	0.10 ± 0.02 a	0.10 ± 0.01 a
γ-Amino-n-butyric acid	0.52 ± 0.02 a	0.49 ± 0.01 a	0.44 ± 0.02 b	0.49 ± 0.02 a	0.43 ± 0.03 b	0.44 ± 0.01 b
Ethanolamin	0.22 ± 0.01 a	0.17 ± 0.01 b	0.19 ± 0.01 b	0.21 ± 0.01 ab	0.20 ± 0.01 a	0.21 ± 0.03 ab
Hydroxylysine	ND	ND	ND	ND	ND	ND
l-Ornithine	0.02 ± 0.01 a	0.02 ± 0.01 a	0.01 ± 0.01 a	0.01 ± 0.01 a	0.02 ± 0.01 a	0.02 ± 0.01 a
1-Methyl-l-histidine	ND	ND	ND	ND	ND	ND
3-Methyl-l-histidine	ND	ND	ND	ND	ND	ND
l-Anserine	ND	ND	ND	ND	ND	ND
l-Carnosine	ND	ND	ND	ND	ND	ND
Hydroxy proline	0.09 ± 0.02 ab	0.08 ± 0.01 b	0.06 ± 0.02 b	0.11 ± 0.01 a	0.09 ± 0.01 ab	0.08 ± 0.02 ab
Sub-total	2.59	2.63	2.12	2.47	2.34	2.35
Total Free Amino Acid	18.37	19.40	14.35	16.63	16.61	16.77

^1^ Samples are defined in Table 1. ^2^ Values are expressed as mean ± standard deviation of two replicates. Values followed by different letters in the same row are significantly different (*p* < 0.05, Tukey test). ^3^ Non-detectable.

## Data Availability

All the data generated in this experiment are presented in the manuscript.

## Sample Availability

Samples of the soybean sprouts are available from the authors.
